# MicroRNA Regulation of Epithelial to Mesenchymal Transition

**DOI:** 10.3390/jcm5010008

**Published:** 2016-01-14

**Authors:** Mohammed L. Abba, Nitin Patil, Jörg Hendrik Leupold, Heike Allgayer

**Affiliations:** Department of Experimental Surgery, Center for Biomedicine and Medical Technology Mannheim (CBTM), Medical Faculty Mannheim, Ruprecht Karl University of Heidelberg, Ludolf-Krehl-Str. 6, 68135 Mannheim, Germany; Mohammed.Abba@medma.uni-heidelberg.de (M.L.A.); Nitin.Patil@medma.uni-heidelberg.de (N.P.); joerg.leupold@medma.uni-heidelberg.de (J.H.L.)

**Keywords:** microRNAs, MET, cancer, EMT, transcription factor

## Abstract

Epithelial to mesenchymal transition (EMT) is a central regulatory program that is similar in many aspects to several steps of embryonic morphogenesis. In addition to its physiological role in tissue repair and wound healing, EMT contributes to chemo resistance, metastatic dissemination and fibrosis, amongst others. Classically, the morphological change from epithelial to mesenchymal phenotype is characterized by the appearance or loss of a group of proteins which have come to be recognized as markers of the EMT process. As with all proteins, these molecules are controlled at the transcriptional and translational level by transcription factors and microRNAs, respectively. A group of developmental transcription factors form the backbone of the EMT cascade and a large body of evidence shows that microRNAs are heavily involved in the successful coordination of mesenchymal transformation and *vice versa*, either by suppressing the expression of different groups of transcription factors, or otherwise acting as their functional mediators in orchestrating EMT. This article dissects the contribution of microRNAs to EMT and analyzes the molecular basis for their roles in this cellular process. Here, we emphasize their interaction with core transcription factors like the zinc finger enhancer (E)-box binding homeobox (ZEB), Snail and Twist families as well as some pluripotency transcription factors.

## 1. Epithelial to Mesenchymal Transition (EMT)

Epithelial cells are characterized by the presence of regular cell-cell contacts and adhesion to the surrounding cellular fabric, preventing the detachment of individual cells [1], as opposed to mesenchymal cells which do not form such intracellular contacts and have irregular cell shapes. As the term denotes, EMT is the transdifferentiation of polarized immotile epithelial cells to motile mesenchymal cells. The process encompasses a form of epithelial plasticity that is characterized by both morphological and molecular changes in epithelial cells [2,3,4,5,6].

Physiologically, the process of EMT occurs during embryonic development and during tissue repair, allowing for the differentiation of cells and remodeling of tissues; however, EMT is also integral to a number of pathological settings including fibrosis and cancer progression [7,8,9,10]. EMT is not a one-way street as a reversal of the process from a mesenchymal to an epithelial state; mesenchymal to epithelial transition (MET) occurs in many systems [11]. Moreover, EMT has also come to be recognized as not being an all or nothing phenomenon with epithelial and mesenchymal states at opposite poles, but rather as a spectrum with a hybrid epithelial/mesenchymal intermediate [12,13,14]. Arguably, this intermediate state, also referred to as partial or incomplete EMT, is seen more as the norm than the exception and represents the EMT phenotype observed during collective migration of neural crest cells in amphibians [15], in Drosophila metamorphosis [16], and at the tumor invasive fronts of several cancers [17,18,19], to mention a few examples.

**Figure 1:**
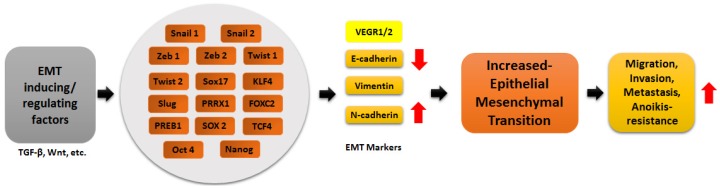
Schematic representation of EMT de-regulating transcription factors and the major key players in the regulation of EMT (down regulation is shown by red downward arrows and up regulation by red upward arrows).

Although the underlying molecular mechanisms that define the pathological and physiological activities of EMT in distinct cellular contexts likely intersect, the diversity of biological outcomes engendered by EMT is nonetheless highly specialized [20,21]. In cancer, particularly, EMT enables epithelial cells to acquire the abilities to invade, resist apoptosis, and to disseminate into distant organs [22,23,24,25,26] (Figure 1).EMT is activated and perpetuated in response to appropriate paracrine signals emanating principally from stromal cells comprising fibroblasts, myofibroblasts and mesenchymal stem cells, amongst others. These stromal cells secrete an array of heterotypic signals that include growth factors like transforming growth factor (TGF)-β, vascular endothelial growth factor (VEGF), hepatocyte growth factor (HGF), epidermal growth factor (EGF), fibroblast growth factor (FGF), platelet-derived growth factor (PDGF) and epidermal growth factor (EGF) leading to the activation of signaling cascades driven by these molecules. Other important signaling cascades important in driving EMT include the Wnt, Notch, Sonic hedgehog pathways [24,27]). Importantly, the EMT-driven metastatic cascade often involves the coordinated interplay of a number of key players that act concertedly to drive tumor dissemination. Our group recently identified a novel network that combined a transcriptional repressor, a histone methyltransferase, an ubiquitin protein ligase and three miRNAs interacting together to foster metastasis [28].

The molecular program that drives EMT is orchestrated primarily by a set of multivalently acting transcriptional factors, including zinc finger enhancer (E)-box binding homeobox 1, (ZEB1), survival of motor neuron protein interacting protein 1 (SIP1) [29,30,31,32,33,34], the zinc finger proteins, SNAI1 (SNAIL) and SNAI2 (SLUG) [30,35,36,37,38] as well as the twist-related protein 1 (TWIST1) [39]. These transcriptional regulators are expressed in various combinations in a number of malignant tumor types and have been shown in experimental models of carcinoma formation to be causally important for programming invasion and even metastasis, when ectopically expressed. Moreover, these transcription factors regulate one another as well as an overlapping set of target genes, with current evidence pinning their involvement in all steps of the invasion-metastasis cascade with the exception of metastatic colonization [40,41]. These transcription factors act to concertedly modulate a loss of adherens junctions, degrade the extracellular matrix and enhance cellular motility, almost always accompanied by the morphological transformation of cells to a spindle-like shape [37,42,43].

## 2. MicroRNAs

MicroRNAs (miRNAs, miRs), a large group of small non-coding RNAs, function by binding to the three prime untranslated region (3′UTR) of messenger RNA (mRNA) molecules, triggering the formation of a complex which, in mammals, results in posttranscriptional repression of the bound mRNA, either by mRNA degradation or inhibition of mRNA protein translation. Overwhelming evidence in literature, as well as experimental studies, have shown miRNAs to be potent regulators of normal cellular physiology. Their effect has been demonstrated in virtually all organ systems and cell types, and their aberrant expression is implicit to several pathological processes and diseases including inflammation, apoptosis, heart failure, Alzheimer’s disease, osteoporosis, diabetes and cancer [44,45,46,47,48,49,50,51,52,53,54,55,56,57,58,59,60]. Their short nucleotides (nt) sequence (22 nt) and even shorter seed sequence (~7 nt) that is required for target binding makes miRNAs “adulterous” molecules, potentially capable of regulating several mRNAs simultaneously [61,62,63]. In this context, particular miRNAs /miRNA families have been found to singularly regulate cellular networks and signaling cascades, making them “master regulators” of these processes [28,64,65,66,67,68,69].

Expectedly, miRNAs also regulate EMT. They do so by interacting with the pool of critical molecules that are involved in engineering EMT, modulating their expression and consequently, their function [2,61,62,63,64,65,70,71,72,73,74,75]. The regulated molecules are many and varied, as EMT itself is complex, and they include mesenchymal proteins, N-cadherin, vimentin, fibronectin, the cytoskeletal and adherens junction proteins including E-cadherin, cytokeratin and occludin but, importantly, the transcription factors that are at the core of the EMT process [76]. This review focusses on the miRNA regulation of the transcription factors involved in EMT (Figure 1).

## 3. Transcription Factors in EMT Regulation

### 3.1. The ZEB Family

The zinc finger E-box binding homeobox (ZEB) family comprises two members, ZEB1 and ZEB2 (also commonly known as smad interacting protein 1 (SIP1)). Their genes are located on the short arms of chromosomes 10 and 2, respectively, and both encode zinc finger transcription factors.These zinc finger proteins are characterized by the presence of two zinc finger clusters at each end of a central homeodomain. ZEB factors also contain multiple independent domains which interact with other transcriptional regulators [11,77,78,79]. ZEB1 and ZEB2 have overlapping, but still distinct, patterns of expression, and they trigger EMT through a combination of repression of epithelial and activation of mesenchymal proteins [2,37,70,79,80,81,82]. Both ZEB factors repress E-cadherin, tight junction protein 3 (TJP3), claudin 4, plakophilin 2, desmoplakin and connexins 26 and 31 [83,84,85,86]. Similarly, both proteins enhance vimentin, N-cadherin and matrix metalloproteinases (MMPs)-1 and -2. ZEB1 also suppresses crumbs 3, lethal giant larvae homolog 2 (LLGL2) and plakophilin 3 [78,87,88,89,90]. By being able to suppress a variety of cell junction type proteins as well as foster mesenchymal properties, ZEB proteins are powerful modulators of EMT.

The miR-200 family, made up of five members, miRs-200a, -200b, -200c, -429, and -141, plays a pivotal role in the regulation of both ZEB transcription factors. A number of reports, all published within weeks of each other, concurred and confirmed the significant role that the miR-200 family members played in maintaining the epithelial phenotype as a result of keeping the ZEB transcription factors in check [91,92,93,94].

In the first of these studies, the expression of 207 miRNAs in the 60 cell lines of the National Cancer Institute’s drug screening panel (NCI60), subcategorized into cell lines with epithelial and mesenchymal phenotypes, identified the miR-200 family as a strong marker for cells that express E-cadherin but lack expression of vimentin [94]. They found miR-200 to directly target the mRNA of the E-cadherin transcriptional repressors ZEB1 and ZEB2 [94]. Korpal and colleagues obtained similar results using NMuMG murine mammary gland epithelial cells induced to undergo EMT with transforming growth factor beta 1 (TGFβ) [93]. Using a slightly different method for EMT induction, Gregory *et al.* delineated the miRNA profiles of wild type canine MDCK (epithelial) and tyrosine phosphatase, non-receptor type 14 (PTPN14) stably transfected MDCK (mesenchymal) cells and observed a significant down regulation of all miR-200 family members, with subsequent 3′UTR luciferase assays, mRNA and protein quantification all showing a significant down regulation of the ZEB proteins, especially upon transfection with miRs-200a and -b. [92]. An extra layer of intricacy was added to the equation when ZEB1 was found to directly suppress transcription of miR-141 and miR-200c, orchestrating a miRNA-mediated double negative feedback loop that stabilized EMT and promoted cancer cell invasion [91]. A myriad of reports have since then validated and re-validated the relationship between the miR-200 family and the ZEB transcription factors in different cell lines, disease types and experimental conditions. Moreover, a cocktail of miRNAs sometimes act together to reinforce the EMT phenotype, a prominent example being the synergistic effects of miR-218 and miR-200 in the regulation of ZEB2 [28].

A higher switch for the activation of the miR-200 family was unraveled when the tumor suppressor p53 was identified as a potent transactivator of a number of miRNAs that included the miR-200 and miR-192 families [95]. Subsequently, p53 was shown to suppress EMT by repressing the expression of ZEB1 and ZEB2. Additionally, the miR-192 family members also repressed ZEB2 expression [95]. Furthermore, miR-130b, another miRNA regulated by p53 also impacts EMT, but in this case, acting via ZEB1. Dong and colleagues were able to show that ectopic expression of p53 mutants repressed the expression of miR-130b and triggered ZEB1-dependent EMT and cancer cell invasion [96]. Loss of an endogenous p53 mutation in endometrial cancer cells increased the expression of miR-130b, attenuating the expression of ZEB1 and subsequently enhancing an epithelial phenotype [96].

Other miRNAs implicated to interact with ZEB transcription factors include miR-139-5p which was found to interact with both factors in hepatocellular carcinoma (HCC), and its suppression promoted EMT, migration, and invasion in Hep3B and SMMC7721 cells [97]. In breast cancer cells, miR-205 was also discovered to directly target ZEB1 and ZEB2; in this case, however, the polycomb ring finger protein 2 (Mel-18) was found to increase miR-205 transcription through the inhibition of DNA methyltransferase-mediated DNA methylation of the miR-205 promoter [98]. Interestingly, miR-205 was also identified as a very significantly upregulated miRNA in esophageal squamous cell carcinoma (ESCC) affecting cell migration and invasion and also targeting ZEB2, but contrary to the norm, was found to be elevated in these tumor cells, although the authors still project it as a tumor suppressor miRNA [99].

Some miRNAs which modulate EMT have been found to interact with just one of the ZEB transcription factors as highlighted below. For instance, in bladder cancer, the expression miR-23b was used to distinguish normal and bladder cancer tissues and high expression of this miR-23b correlated positively with higher overall survival of bladder cancer patients [100]. ZEB1 was found to be the direct target of miR-23b and responsible for promoting bladder cancer cell migration and invasion [100].

*In vitro* assays showed ZEB1 as a new direct target of miR-150 and that miR-150 induced mesenchymal–epithelial transition (MET). MET-like changes in TE-8 ESCC cells mediated through ZEB1 degradation were able to inhibit tumorigenicity and tumor growth in a mouse xenograft model [101]. Moreover, miR-150 expression was significantly lower in cancer tissues compared to adjacent non-cancerous tissues and correlated with tumor size, lymph node metastasis, lymphatic invasion, venous invasion, clinical staging, and poor prognosis [101]. Still, miR-150 has been reported to also be downregulated in human epithelial ovarian cancer (EOC) tissues and patients’ serum compared to normal controls, and ectopic expression of miR-150 could efficiently inhibit cell proliferation, invasion and metastasis by suppressing the expression of ZEB1 [102].

In an analysis of 71 colorectal cancer patients, miR-147 was identified as highly negatively-correlated with an EMT gene expression signature score and postulated to reverse EMT (MET). MiR-147 was found to primarily act by increasing the expression of cadherin type 1 (CDH1) and decreasing that of ZEB1, which it targets directly, resulting in the inhibition of cell motility and invasion. Additionally, miR-147 was able to dramatically reverse the native drug resistance of the HCT116 colon cancer cell line to Gefitinib [103].

Qu and colleagues discovered that miR-33b expression was dramatically decreased in lung adenocarcinoma cell lines and tissues, and this reduced expression was associated with tumor lymph node metastasis mediated in part by the binding of miR-33b to the ZEB1 3′UTR region inhibiting ZEB1 expression [104].

Using a strategy that included a red fluorescent promoter reporter gene carrying the vimentin promoter together with additional morphological experiments, Yanaka and colleagues screened a 328-miRNA library in search of EMT inducing miRNAs and identified miR-544a as the most potent in gastric cancer cells. They demonstrated that the overexpression of miR-544a induced the expression of ZEB1, but also that of vimentin, and SNAI1, while reducing CDH1 expression accompanied by an EMT phenotype. Subsequently, they showed that the reduction of CDH1 and AXIN2 by miR-544a also activated the wingless (WNT) signaling pathway by stabilizing β-catenin in the nucleus [105].

The tumor suppressor p21 functions downstream of p53 and has been shown to directly induce the transcription of certain EMT miRNAs. For example, in investigating if p21 was involved in EMT, one group sequenced and compared RNA reads from isogenic p21^(+/+)^ and p21^(−/−)^ cells and identified the miR-183-96-182 cluster, in addition to the well documented miR-200 family, to be downregulated in p21-deficient cells. They found that miR-183 and miR-96 repressed common targets that included ZEB1, as well as SLUG, integrin beta 1 (ITGB1), and Kruppel-like factor 4 (KLF4), and the restoration of miR-183, or miR-96 in p21^(−/−)^ cells inhibited EMT, cell migration, and invasion. Interestingly, they found that p21 forms a complex with ZEB1 at the miR-183-96-182 cluster promoter to inhibit transcriptional repression of the cluster by ZEB1 [106].

The rate of mRNA decay is determined by *cis*-acting sequence elements contained within individual mRNAs that serve as binding sites for *trans*-acting factors that may positively or negatively impact the mRNA degradation process [107]. One of the best characterized *trans*-acting factors is the heterogeneous nuclear ribonucleoprotein D (HNRED) or AUF1 family of proteins, which bind to, and stabilize, certain mRNAs. Al-Khalaf and Aboussekhra discovered that AUF1 binds the 3′UTR of the ZEB1 mRNA and reduces its turnover. Furthermore, they found that miR-141 and miR-146b-5p were able to bind the 3′UTR of AUF1, leading to its down-regulation and in the process positively enhancing the epithelial phenotype [108].

Other miRNAs that interact with ZEB1 include miR-101, which was found to significantly inhibit the TGF-β1-induced EMT in hepatocytes [109]. Individual examples pertaining to ZEB2 include, for example, miR-153, which was shown to directly target ZEB2 in human EOC in the process suppressing EMT as well as invasion [110]. Others include miR-338-3p which was also discovered to inhibit migration and invasion of gastric cancer cells *in vitro* by interacting with ZEB2 together with the metastasis-associated in colon cancer-1 (MACC1) mRNA [111]. In bladder cancer, miR-145 was found to also regulate ZEB2, but, interestingly, the long non-coding RNA taurine up-regulated 1 (TUG1) was found to modulate the levels of miR-145 by acting as a competing endogenous RNA (ceRNA) for miR-145 [112]. This miRNA was found to be further active in prostate cancer cells, where it was found to limit invasion, migration, EMT, and the stemness of prostate cancer cells via the repression of ZEB2. Remarkably, ZEB2 was also found to directly repress the transcription of miR-145, establishing a double-negative feedback loop between ZEB2 and miR-145 with strong implications for prostate cancer metastasis [113].

In colorectal cancer, miR-132 was also found to be a relevant mediator of EMT as a result of its targeting of ZEB2, and was found to be of added prognostic value clinically, as patients with low expression of miR-132 tended to have worse disease-free survival than patients with high expression of miR-132 [114]. The mechanistic effects of miR-132 in EMT were also observed in human non-small cell lung cancer (NSCLC) where ZEB2 was also the major target [115].

Still, miR-138 was identified as another regulator of ZEB2. Working on head and neck squamous cell carcinoma cell lines, Liu and colleagues demonstrated that miR-138 regulated EMT not only by targeting ZEB2 but also via the direct targeting of vimentin mRNA and enhancer of zeste homologue 2 (EZH2), an epigenetic regulator which modulates the silencing of E-cadherin [116].

**Figure 2:**
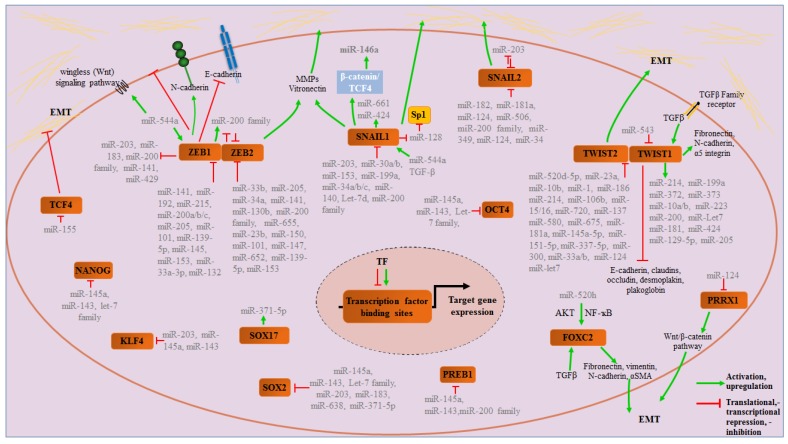
Overview of significant miRNAs involved in EMT regulation. This is specific to transcription factors only and includes microRNAs targeting transcription factors and microRNAs enhanced or repressed by transcription factor activity. An up-regulation/activation is shown by green arrows and translational/transcriptional repression, or -inhibition by red truncated lines.

As cellular proteins, ZEB transcription factors are also transactivated by other transcription factors; for instance, ZEB2 is regulated by the GATA family transcriptional repressor tricho-rhino-phalangeal syndrome type 1 (TRPS1). TRPS1 is directly regulated by miR-221 and miR-222, and the expression of these two miRs was found to induce EMT by decreasing the expression of epithelial-specific genes and enhancing those of mesenchymal-specific genes, accompanied by increased cell migration and invasion [117,118]. This example underscores the intricacy of miRNA regulation of EMT (Figure 2).

### 3.2. The Snail Family (Snai1, Snai2 and Snai3)

The Snail family of zinc-finger transcription factors comprise SNAI1 (often designated as SNAIL), SNAI2 (also known as SLUG), and SNAI3 (also known as SMUC). They occupy a central role in EMT, as well as embryonal morphogenesis [11,81,119,120,121,122]. All three transcription factors contain a single C-terminal zinc finger cluster that binds to E-boxes in the regulatory regions of target genes and an N-terminal SNAG (Snail/Gfi) domain that is important for repression in mammalian cells [123]. Upon binding to the E-box, Snail family members act as transcriptional repressors and recognize a core consensus binding motif that is identical to that recognized by the basic helix-loop-helix (bHLH) transcription factors [124].

SNAI1 and SNAI2 are the dominant players orchestrating EMT, and there is little literature about SNAI3 and EMT. Unlike the ZEB family of transcription factors, SNAI1 and SNAI2 appear to have no overlapping roles in EMT as evidenced by the non-overlapping mRNA and miRNA clusters in both expression profiling types [125,126,127]. Nonetheless, Moreno-Bueno and colleagues in their attempt to elucidate mRNAs regulated by these factors identified collagen type V alpha 2 (COL5A2), moesin (MSN), TIMP metallopeptidase inhibitor 1 (TIMP1), serpin peptidase inhibitor, clade H (heat shock protein 47) member 1, (collagen binding protein 1) (SERPINH2), WAS/WASL interacting protein family member 1 (WASPIP), collagen type I alpha 2 (COL1A2), integrin alpha 5 (fibronectin receptor, alpha polypeptide) (ITGA5), caldesmon 1 (CALD1), serpin peptidase inhibitor clade E (nexin, plasminogen activator inhibitor type 1) member 1 (SERPINE1), collagen type III alpha 1 (COL3A1) and secreted protein acidic cysteine-rich (osteonectin) (SPARC) as commonly up-regulated and CDH1, secreted phosphoprotein 1 (SPP1), fibroblast growth factor binding protein 1 (FGFBP1), keratin 19 type I (KRT19), tetraspanin 13 (TSPAN13), integrin beta 6 (ITGB6) as commonly downregulated by Snai1 and Snai2 [125].

Likewise, in the search for a core miRNA-EMT signature, Diaz-Martin *et al.* induced EMT in the canine epithelial MDCK cell line using the major EMT inducing transcription factors comprising the Snail, ZEB and Twist families, and lysyl oxidase-like 2 (LOXL2) individually followed by comparison of differentially regulated miRNAs across the board looking for commonalities [127,128,129]. Interestingly, they found members of the miR-200 family (miRs-200a, -200b, -200c, and -141), the miR-182 cluster (miRs-182, -183), miRs-18a, -18b, -486-5p, -486-3p, -31, -203, -100, -223, -99a, -375 and-133a as significantly deregulated in both situations. With the exception of miR-133a, whose expression was upregulated, all of the others were significantly downregulated by these transcription factors, including SNAI1 and SNAI2 [128,129]. This invariably not only indicates the central role that miRNAs in general, but that these miRNAs in particular, play in orchestrating EMT, but also to the fact that the core EMT machinery has an essential function in modulating miRNA expression, which makes them key effector molecules of the process (Figure 2).

A number of miRNAs have been documented to suppress the expression of SNAI1, with a few members of the miR-30 family showing significance. We found the expression of miR-30a to be inversely proportional to the invasive potential of various NSCLC cell lines correlating negatively with N-cadherin expression. Forced expression of miR-30a was able to alter cell morphology and suppress migration and invasion *in vitro*. This was paralleled by a repression of SNAI1, which was shown to be its direct target. Moreover, distant metastases to the lungs and liver were also suppressed in the presence of miR-30a in the chicken embryo model [130]. A similar phenomenon was demonstrated in hepatocellular carcinoma cell lines [131].

In their bid to elucidate the roles of p120 and E-cadherin in epithelial cell behavior, Kourtidis and colleagues showed that miR-30b was critical to the suppression of cell transforming markers that included Snai1, and the levels of miR-30b were regulated by pleckstrin homology domain containing family A member 7 (PLEKHA7), a p120 binding partner and an essential component of the cadherin complex [132]. A direct regulation was, however, not shown.

Another implicated miRNA group is the miR-34 family, a p53 regulated set. Kim and colleagues demonstrated that p53 loss-of-function or mutations promoted EMT by de-repressing SNAI1 protein expression and activity in multiple cancer cell lines. This was attributed to a decrease in miRNA-34 levels (miR-34a, miR-34b, and miR-34c), which suppressed SNAI1 directly by binding to a highly conserved region of its 3′UTR. The EMT effect was reinforced by the repression of other regulatory molecules, including β-catenin, LEF1, and Axin2 all of which contained miR-34 binding sites that were also sensitive to miR-34 dependent regulation [133].

Using a miRNA array in squamous cell carcinoma of the tongue cell lines whereby EMT was induced with TGFβ in one pair and the metastasis mesenchymal derivative of the primary cell line in the other, miR-153 was identified as significantly repressed in cells undergoing EMT. Ectopic expression of miR-153 in mesenchymal-like cells resulted in an epithelial transformation with decreased invasive abilities and to Snai1 suppression [134]. Similar results were obtained in gastric cancer where miR-153 was able to suppress migration and invasion by inhibiting SNAI1-induced EMT and also serve as an independent prognostic marker for predicting survival of gastric cancer patients [135] as was the case in pancreatic ductal adenocarcinoma (PDAC) [136].

Other significant miRNAs that impact SNAI1 expression include miR-199a which was found to increase the protein levels of claudin-1 in both TGF-β1-treated and -untreated cells in part by decreasing the protein level of SNAI1, a repressor of claudin-1 [137].

SNAI2 has its own unique set of regulating miRNAs that include miR-506 [138,139,140], miR-124 [141] and miR-181a [142]. Some miRNAs like miR-203 targets both SNAI1 and SNAI2 [143,144].

### 3.3. The Twist Family (TWIST1 and TWIST2)

The Twist family of basic helix-loop-helix transcription factors comprising TWIST1 (202 amino acids) and the smaller TWIST2 (also known as Dermo-1) (160 amino acids) play key roles in embryonic development. Both proteins have a conserved C-terminal Twist box interaction domain and basic Helix Loop Helix motif which is able to recognize E-box responsive elements (which binds to CANNTG region). Twist proteins act as either transcription repressors or activators, depending on the cellular context [145]. They are able to form homo- and heterodimers with each other [146,147,148,149] and to directly interact with a large set of transcription factors. The Twist factors are overexpressed in most in human cancers and in most cases correlate with high tumor grade, invasiveness, and metastasis [150,151,152,153,154,155,156].

As with the other transcription factors, several miRNAs are involved in the EMT modulating properties of the Twist proteins.

One of the upstream activators of TWIST is c-Src, which itself is activated by CD44 a cell-surface glycoprotein involved in cell-cell interactions, cell adhesion and migration. C-Src was found to increase Twist phosphorylation, promote its nuclear translocation and subsequently lead to the stimulation of miR-10b expression which is directly regulated by Twist. Inhibition the c-Src/TWIST axis causes down-regulation of Ras homolog gene family members RhoA/RhoC expression, impairment of tumor cell invasion and mitigation of the metastatic properties of MDA-MB-231 breast cancer cells [157]. Still, another group was able to demonstrate that miR-10b expression in breast cancer cells could be suppressed by wingless-type MMTV integration site family, member 1 (WNT1), inducible signaling pathway protein 2 (WISP2), a member of the WISP protein subfamily that acts by inhibition of TWIST1 expression [158].

In response to hypoxia, hypoxia inducible factor 1 α (HIF1α) is activated, leading to a surge in TWIST1 levels accompanied by the induction of miR-372/373 expression. The miR-372/373 targets reversion-inducing-cysteine-rich protein with kazal motifs (RECK) resulting in enhanced malignant cell behavior [159]. Taking a hint from similarly regulated homologues in mice, Yin and colleagues investigated a possible regulatory role of Twist1 in expression of the hsa-miR-199a/hsa-miR-214 cluster in human EOC cells and showed that TWIST1 induces the expression of the hsa-miR-199a/hsa-miR-214 cluster in these cells. Moreover, knowing that miR-199a directly regulates the expression of the inhibitor of kappa light polypeptide gene enhancer in B-cells, kinase beta (IKKβ), they proceeded to demonstrate that knocking down TWIST1 increased the levels of IKKβ significantly [160].

In dissecting the miRNAs that were differentially expressed in gastric cancer, Li and colleagues identified miR-223 to be overexpressed in metastatic gastric cancer cells only, and stimulated migration and invasion in non-metastatic gastric cancer cells. They discovered that miR-223 was induced by TWIST via binding to an E-box located in its core promoter, then binding to the 3′UTR of erythrocyte membrane protein band 4.1-like 3 (EPB41L3) and suppressing its translation [161].

Additional examples of miRNAs that are induced by TWIST1 include miR-181a [162,163] and miR-424, which were discovered to be upregulated early during a TWIST1 or SNAIL1-induced EMT, and cause the expression of mesenchymal genes without affecting epithelial genes [164].

As stated earlier, TWIST is also able to act as a transcriptional repressor, and such an effect was evident with miR-200 and miR-205. *TWIST1* mRNA levels were found to be higher in cells with very low miR-200/miR-205 expression. Subsequently, an interaction with the promoters of miR-200/miR-205 was shown in HU609 cells, resulting in a stronger induction of gene expression then reported for the valid TWIST1 repression target, CDH1 [165].

In OSCC, let-7d expression was shown to negatively correlate with Twist and Snail expression in clinical samples as well as primary cultures. Scavenging endogenous let-7d in cultured cells with a sponge resulted in enhanced expression of TWIST, mesenchymal morphology, increased migration and colony formation [166]. In a much broader approach, Haga and Phinney decided to investigate the imprinted delta-like 1 homolog-deiodinase, iodothyronine, type III (DLK1-DIO3) region containing several miRNAs. They identified seven miRNAs, miRs-300, -382, -494, -495, -539, -543, and -544, located in this region that serve as tumor suppressors by cooperatively repressing an EMT signaling network comprising TWIST1, BMI1 polycomb ring finger proto-oncogene, ZEB1/2, and the miR-200 family. Particularly, they were able to show that miRs-300, -539 and -543 significantly repressed TWIST1 [167]. Nairismägi *et al.* analyzed the translational regulation of TWIST1 using luciferase reporter assays in a variety of cell lines and found miR-145a-5p, miR-151-5p and miR-337-3p to be able to individually or in combination significantly repress Twist1 translation. They confirmed their findings with both exogenous and endogenous miRNAs. Twist suppression resulted in a decreased migratory potential of murine embryonic fibroblast cells [168,169]. The same group previously had looked at the *TWIST1* 3′UTR and identified miR-580 and two cytoplasmic polyadenylation elements, cytoplasmic polyadenylation element binding protein-1 and -2 (CPEB1, CPEB2), additional regulators of TWIST1 expression in MCF-10A cells [168].

A statistical analysis of 105 cases of primary human breast cancer demonstrated that decreased expression of miR-720 correlated with lymph node metastasis, and overexpression of this miRNA in breast cancer cells inhibited cell migration and invasion *in vitro* and *in vivo*. TWIST1 was identified as a direct functional target of miR-720 [170]. In addition, in HCC cells, miR-675 was found to mediate epithelial reprogramming through inhibition of TWIST1 expression, and miR-675 overexpression resulted in altered cellular morphology, reduced invasive potential, and increased anchorage-independent growth capacity and TWIST1 was identified as a direct target of this miR [171].

Other documented direct inhibitors of TWIST1 include miR-520d-5p [172], miR-137 [173], miR-33a [174,175], miR-186 [176] and miR-1-1 [177].

### 3.4. Pluripotency Transcription Factors

Stemness and EMT have often been observed in the same context, and there are a number of reports which have shown that these two processes are intertwined. Robert Weinberg’s lab demonstrated that it was possible to induce stem cell features in somatic immortalized human mammary epithelial cells (HMLEs) by overexpressing either TWIST or SNAI1 [178], with similar results being obtained by Morel and colleagues [179]. Conversely, the EMT-activator ZEB1 has been shown to promote tumorigenicity by repressing stemness-inhibiting miRNAs, miR-200c, miR-203 and miR-183 inclusive [180].

The transcription factors comprising SOX2, OCT-3/4, KLF4, NANOG and c-MYC form a core pluripotency network which governs the preservation of the pluripotent status quo, with OCT4, NANOG, and SOX2 shown to contribute to the reprogramming of somatic cells into an ESC-like state [61].

The SOX (SRY-related HMG-box) family of transcription factors is involved in the regulation of embryonic development, stemness and cell differentiation. A total of 20 Sox genes are present in the mammalian genome [181], and target gene selectivity by different Sox factors is realized through the differential affinity for particular flanking sequences next to consensus Sox sites, homo- or heterodimerization among Sox proteins, posttranslational modifications of Sox factors, or interaction with other cofactors [182,183,184]. The SOX transcription factors normally synergize with SNAI1 or SNAI2 in driving EMT and/ or cell invasion, and the prominent members which have been implicated in EMT include SOX2, SOX9 and SOX17 [185].

The Krüppel-like transcription factors are zinc finger proteins that activate and suppress target gene transcription. The family members share three highly conserved classical Cys2/His2 zinc fingers which are located at the carboxyl terminus of the protein and enable Klfs to bind to related GC- and CACCC-boxes of DNA. KLF transcription factors are involved in the regulation of many developmental processes [186,187]. KLF4 appears to be the only member implicit to EMT.

NANOG is a DNA binding homeobox transcription factor involved in embryonic stem cell proliferation, renewal, and pluripotency and is expressed in the founder cells of the early mouse embryo, being the reason why it was named after the mythological Celtic land of the ever young, Tir nan Og, by the scientists that first identified its function [188].

Despite the entangled relationship between EMT and the pluripotency transcription factors, most of the existing literature elucidates only indirect or supporting functions for these transcription factors in EMT. As such, the miRNAs which are either regulated by, or regulate, these transcription factors also affect EMT indirectly. For instance, in pancreatic ductal adenocarcinoma, the loss of doublecortin-like kinase1 (DLCK1) results in the enhanced expression of miR-145, let-7 and miR-200. Increased levels of miR-145 results in the decreased expression of OCT4, SOX2, NANOG, KLF4 as well as KRAS and RREB1, whereas the increase in miR-200 culminates in the decreased expression of VEGFR1, VEGFR2 and EMT-related transcription factors ZEB1, ZEB2, SNAIL and SLUG [189]. The same group went on to show that XMD8-92, a kinase inhibitor with anti-cancer activity, inhibited AsPC-1 cancer cell proliferation and tumor xenograft growth via the downregulation of DCLK1 and subsequently enhanced expression of several miRNAs, with the inclusion of miR-143/145 to those previously reported. The affected downstream targets remained the same [190]. Xia and colleagues were able to demonstrate a direct binding of miR-638 to the 3′UTR of *SOX2* with resultant significant suppression of its expression that was associated with a repression of SNAI1, fibronectin and vimentin as well as a concomitant increase in the expression of E-cadherin. It was not clear if SOX2 was responsible for the reversal of the EMT phenotype (MET), or whether miR-638 had other targets that were responsible for this observation [191].

By using ICG-001, a specific CREB-binding protein (CBP)/β-catenin antagonist in Epstein- Barr Virus (EBV) positive nasopharyngeal carcinoma, Chan *et al.* observed a reduction in the cancer-stem-cell-like population of cells that, amongst other outcomes, was associated with an increase in miR-145. They observed that the ectopic expression of miR-145 effectively repressed SOX2 (its direct target) protein expression and inhibited tumor sphere formation. ICG-001-treated cells manifested re-expression of E-cadherin and decreased expression of vimentin after seven days of treatment. In addition, in this case, no direct link between Sox2 and the EMT phenotype was demonstrated [192].

An additional example includes the identification of SOX2 as the direct target of miR-371-5p, whose own expression was influenced by SOX17. The SOX17/miR-371-5p/SOX2 axis demonstrated a significant role in the regulation of EMT (vimentin, N-cadherin, TWIST2 increased; E-cadherin suppressed), stemness and metastasis [193]. The regulation of EMT by miR-371-5p was attributed to modulation of Wnt/β-catenin signaling, as no direct relationship to SOX2 was evident [194].

### 3.5. Other Transcription Factors

Finally, there are a number of other transcription factors linked to EMT, where, however, the literature is thin. These factors also appear to be of significance in orchestrating EMT and include the zinc finger protein 281 (ZNF281) whose expression is induced by SNAI1 and inhibited by miR-34a [195] and the paired-related homeobox protein 1 (PRRX1; PRX-1), a rather recent addition to the EMT inducers, which, unlike other EMT transcription factors, does not concur with the described induction of stem cell-like properties concomitant with Snail-, TWIST-, or ZEB-mediated mesenchymal transitions [196] and which is targeted by miR-124 [197]. Last but not least, a basic helix-loop-helix transcription factor, the transcription factor 4 (TCF4), is directly regulated by miR-155 [198].

## 4. Conclusions

MicroRNAs play crucial roles in EMT, either as effector molecules of the core transcription factors or as modulators of their expression. Judging by the amount of published data, some miRNAs stand out as arguably the master regulators of EMT, most prominently, at present, the miR-200 family. This is not to in any way undermine the significance of the other players, given, as we know, the cell type and context-associated function of most miRNAs. One of the most insightful overviews comes perhaps from the work of Diaz-Martin and colleagues who attempted to decode the core miRNA signature associated with EMT and made an excellent assemblage. Interestingly, newly identified molecules with transcriptional activity, most recently for example, PRRX1 that impact EMT, may prove in future to be as significant as those already known. It remains without question that newer roles will be ascribed to the miRNAs already implicated in EMT or novel proteins will be added to the machinery that drives EMT.
